# Ag nanoparticle-deposited TiO_2_ nanotube arrays for electrodes of Dye-sensitized solar cells

**DOI:** 10.1186/s11671-015-0924-1

**Published:** 2015-05-15

**Authors:** Go Kawamura, Hayato Ohmi, Wai Kian Tan, Zainovia Lockman, Hiroyuki Muto, Atsunori Matsuda

**Affiliations:** Department of Electrical and Electronic Information Engineering, Toyohashi University of Technology, 1-1 Hibarigaoka, Tempaku-cho, Toyohashi, 441-8580 Aichi Japan; School of Materials and Mineral Resources, Engineering Campus, Universiti Sains Malaysia, Seri Ampangan, 14300 Nibong Tebal, Pulau Pinang Malaysia

**Keywords:** Anodization, TiO_2_ nanotube arrays, Ag nanoparticles, Light harvesting, Dye-sensitized solar cells

## Abstract

**Abstract:**

Dye-sensitized solar cells composed of a photoanode of Ag nanoparticle (NP)-deposited TiO_2_ nanotube (TNT) arrays were fabricated. The TNT arrays were prepared by anodizing Ti films on fluorine-doped tin oxide (FTO)-coated glass substrates. Efficient charge transportation through the ordered nanostructure of TNT arrays should be carried out compared to conventional particulate TiO_2_ electrodes. However, it has been a big challenge to grow TNT arrays on FTO glass substrates with the lengths needed for sufficient light-harvesting (tens of micrometers). In this work, we deposited Ag nanoparticles (NPs) on the wall of TNT arrays to enhance light-harvesting property. Dye-sensitized solar cells with these Ag NP-deposited TNT arrays yielded a higher power conversion efficiency (2.03 %) than those without Ag NPs (1.39 %).

**PACS codes:**

06.60.Ei Sample preparation, 81.05.Bx Metals, Semimetals, Alloys, 81.07.De Nanotubes

## Background

Photovoltaics is now taking part in the global movement from fossil fuels to renewable sources. Dye-sensitized solar cell (DSSC) has been recognized as a probable competitor to the widely used but relatively expensive solar cells including silicon and copper indium gallium diselenide (CIGS) due to its lower cost, lower sensitivity to light angle of incidence, and easy fabrication on flexible substrates [1, 2]. The power conversion efficiency of DSSC has now reached around 13 % [3], which is still less than half of the high-efficiency solar cells mentioned above. DSSCs are generally composed of a photoanode consisting of anatase nano-particulate film with thicknesses of >10 μm on a transparent conducting oxide (TCO) glass substrate supporting a monolayer of a dye, a platinum foil or a platinum-coated TCO glass counter-electrode, and an iodide electrolyte between them. The electrons generated in the dye by light irradiation are injected into the anatase nano-particulate film, and then diffuse through the nanoparticle network in the film before reaching to the TCO substrate. The electrons undergo millions of recombination events at defect sites and interfaces between nanoparticles during percolating through the TiO_2_ nano-particulate film. Therefore, an increase in the TiO_2_ film thickness significantly increases electrical resistivity of DSSCs, though thick TiO_2_ films are generally employed to increase the amount of dyes and to enhance light-harvesting ability.

In order to achieve high-energy conversion efficiency of DSSC with thinner TiO_2_ film thickness, the invention of new dyes that strongly absorb sun light, which contains both short- and long-wavelength photons, should be carried out [4]. Alternatively, vertically aligned TiO_2_ architectures such as nanorod and nanotube arrays that offer longer electron diffusion lengths (decreased charge recombination rate) and shorter electron transport time should be fabricated. Among various architectures, the most promising one for solar energy conversion appears to be TiO_2_ nanotube (TNT) arrays prepared through anodization of titanium [5–10]. Remarkably enhanced charge collection efficiency and light scattering in DSSCs fabricated with TNT arrays grown on a Ti foil were reported previously by comparing to DSSCs with conventional TiO_2_ nano-particulate films [6]. The electron diffusion length in TNT arrays was found to be three times longer than nano-particulate films [11]. However, the highest power conversion efficiency of DSSCs with TNT arrays reported so far is only 6.9 %, which is about half of the efficiency of DSSCs with conventional TiO_2_ nano-particulate films [12]. The authors of the paper concluded that the major issue that limited the conversion efficiency in DSSCs with TNT arrays was the low fill factor, which was obtained by the degradation of the fluorine-doped tin oxide (FTO) substrates. This degradation of FTO occurred during long-term heat treatment at high temperature which was necessary to improve crystallinity of TNT arrays with the length of 17.6 μm, which is about five times longer than TNT arrays commonly fabricated by anodization by Ti.

In this work, Ag nanoparticles were deposited on the wall of the TNT arrays on FTO substrates. Ag nanoparticles exhibit localized surface plasmon resonance (LSPR) that strongly absorbs and scatters photons compared to dyes and TiO_2_. The enhanced electric field by LSPR of Ag nanoparticles efficiently excites electrons in neighboring dyes. The photon scattering phenomenon by LSPR works as mirror-like scattering layers, which are often formed to enhance light-harvesting ability of DSSCs [13]. As a result, the optimum length of TNT arrays should be shortened, requiring a shorter heating time at a lower temperature. DSSCs with TNT arrays on FTO were fabricated, and the effects of Ag nanoparticle deposition onto the TNT arrays on the performance of DSSCs were investigated.

## Methods

### TiO_2_ nanotube arrays on FTO

Radio frequency magnetron sputtering of Ti was carried out at 50 W cm^−2^ of power density and 5 mTorr in an Ar gas atmosphere for 5 h to form Ti films with a thickness of 3 μm on FTO glass substrates (10 Ω □^−1^, 85 % transmittance). The Ti deposition rate was estimated to be 8.4 nm min^−1^. The substrates were then dipped into electrolytes of ethylene glycol (EG) with NH_4_F and H_2_O, where the weight ratio of EG:NH_4_F:H_2_O was 0.3:2:97.7. The electrolyte was prepared and used without any conditioning process. Applied voltages of 10–40 V were used for anodizing the Ti film on FTO; anodization duration was also varied from 30 to 60 min to control the tubular pore structures. The samples were washed with 2-propanol and then washed with H_2_O. After drying in air at room temperature, heat treatment at 450 °C with a ramp rate of 3 °C min^−1^ was carried out for 4 h to crystallize TNT arrays without collapsing the tubular structures. The arrays were soaked into 0.3 mM N719 dye (Aldrich) of acetonitrile and butanol-mixed solution for 16 h. Excessively adsorbed dyes were rinsed out by washing with methanol. Ag nanoparticle deposition was done by dipping the arrays into 0.1 M AgNO_3_ aqueous solution, followed by radiating ultra-violet (UV, 365 nm) at 1 mW cm^−2^ for 3 min.

### Structural analyses

A Hitachi S-4800 scanning electron microscope (SEM) was used to study the morphology of TNT arrays. A Rigaku Ultima IV R285S X-ray diffractometer (XRD) was used to examine TiO_2_ crystallinity and Ag deposition. A JEOL JEM-2100 F transmission electron microscope (TEM) equipped with a JEOL 2300 T energy-dispersive X-ray spectroscope (EDX) was used to study the dispersion state of Ag nanoparticles in the TNT arrays. An FEI Quanta focused-ion beam system (FIB) was used to prepare monolith samples with a size of 10 × 4 μm and a thickness of 100 nm for the cross-sectional TEM-EDX analysis. A JASCO V-670 UV-Vis-NIR spectrophotometer was used to investigate the effect of Ag nanoparticle deposition on optical absorbance of the photoanode.

### DSSC fabrication and evaluation

**Fig. 1:**
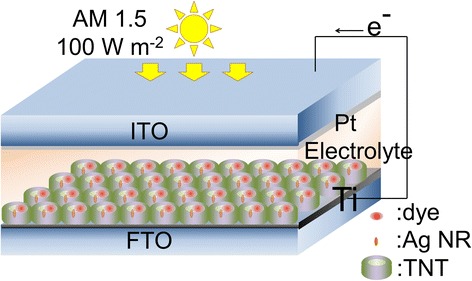
Illustration of fabricated DSSC

The prepared TNT arrays on FTO were used as photoanode. The counter electrodes were prepared by sputtering Pt of a few nanometer thick on indium tin oxide (ITO) glass substrates. The electrolyte for DSSCs was synthesized using acetonitrile mixed with 0.05 M iodine, 0.1 M lithium iodide, 0.6 M 1,2-dimethyl-3-propylimidazolium iodide, and 0.5 M 4-tert-buthylpyridine. After preparing each component as above, spacer films (DU PONT, Himilan) of a thickness of 50 μm, leaving a window of an area of 1 cm^2^, were sandwiched in two electrodes and heated at 220 °C for 15 min. The electrolyte solution was finally injected into the space between two electrodes. The final architecture is illustrated in Fig. 1. The current-voltage characteristics of the DSSC were investigated using a SMU source/measure unit (Asahi spectra) equipped with a HAL-C100 solar simulator (Asahi spectra) composed of a 300-W xenon lamp and an air-mass 1.5 global filter.

## Results and discussion

### Morphology of TiO_2_ nanotube arrays

**Fig. 2:**
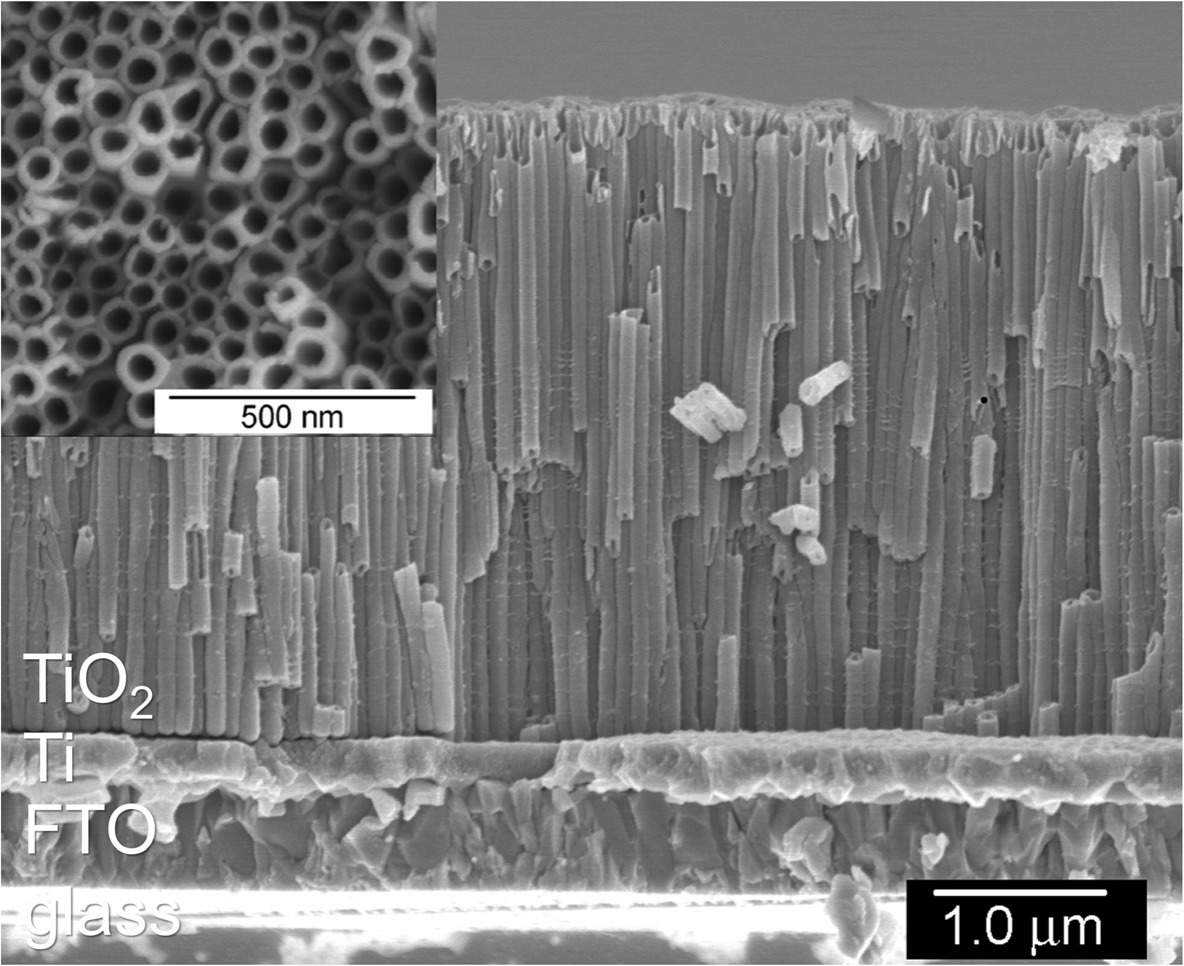
SEM image of as-anodized TNT arrays

Figure 2 shows the SEM images of TNT arrays prepared by anodizing Ti films on FTO at 40 V for 40 min. Generally, debris is formed on the top of TNT arrays after anodization, and they partially close the tubular pore ends. TNT arrays with partially closed ends are less appropriate for DSSC applications, because electrolyte dissolution is limited during generation of electricity. On the other hand, the arrays prepared in this work were obviously debris free. This is presumably because of the low Ti dissolution rate, which depends on the concentration of oxidants (NH_4_F and H_2_O in this work) in electrolytes for anodization [14, 15]. The length of the tubes was measured to be ~3.9 μm and the diameter of the pores was ~51 nm. The Ti film was not fully anodized, resulting in formation of Ti interlayer of ~200 nm between TNT arrays and FTO. The Ti interlayer worked to have good physical and electronic contacts between TNT arrays and FTO.

**Fig. 3:**
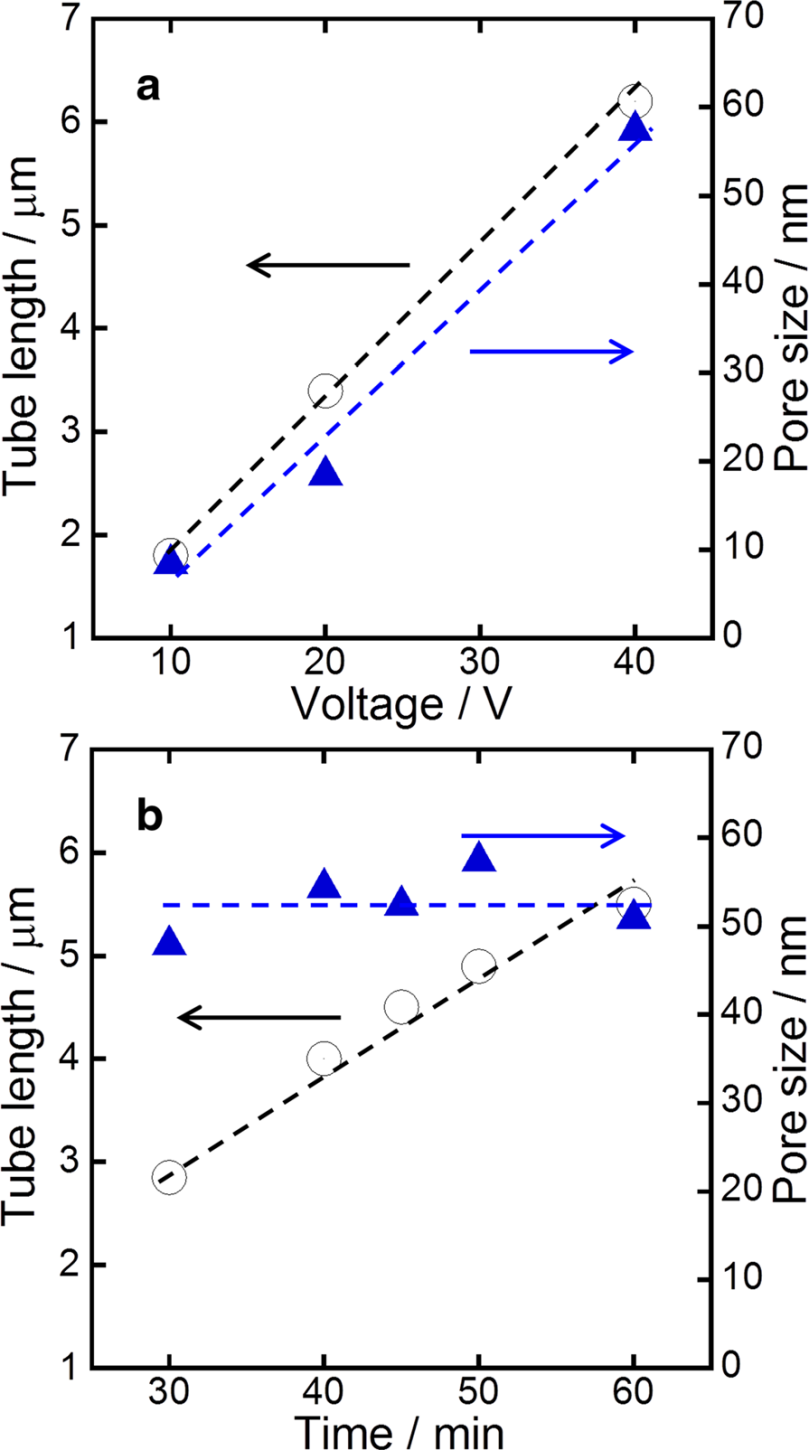
Variation of tube lengths and pore sizes of TNT arrays as functions of applied voltage (**a**) and anodizing time (**b**)

The tube length and pore diameter of TNT arrays were able to control by altering applied voltage and anodization time as shown in Fig. 3. Both the tube length and the pore diameter were increased with increasing applied voltage. Conversely, the pore diameter was not increased when anodization time increased, whereas the tube length was well dependent on the time. Since longer nanotube arrays with smaller pore diameters should possess larger roughness factor, which is the ratio of the real surface area and the flat surface area, they are appropriate for DSSC applications. However, since debris and clump appeared when the tube length was too long and pore diameter was too small. Therefore, TNT arrays with ~3.9 *μ*m tube length and ~51 nm pore diameter were employed for DSSC fabriaction in this work.

### Dispersion of Ag nanoparticles on the wall of nanotube arrays

**Fig. 4:**
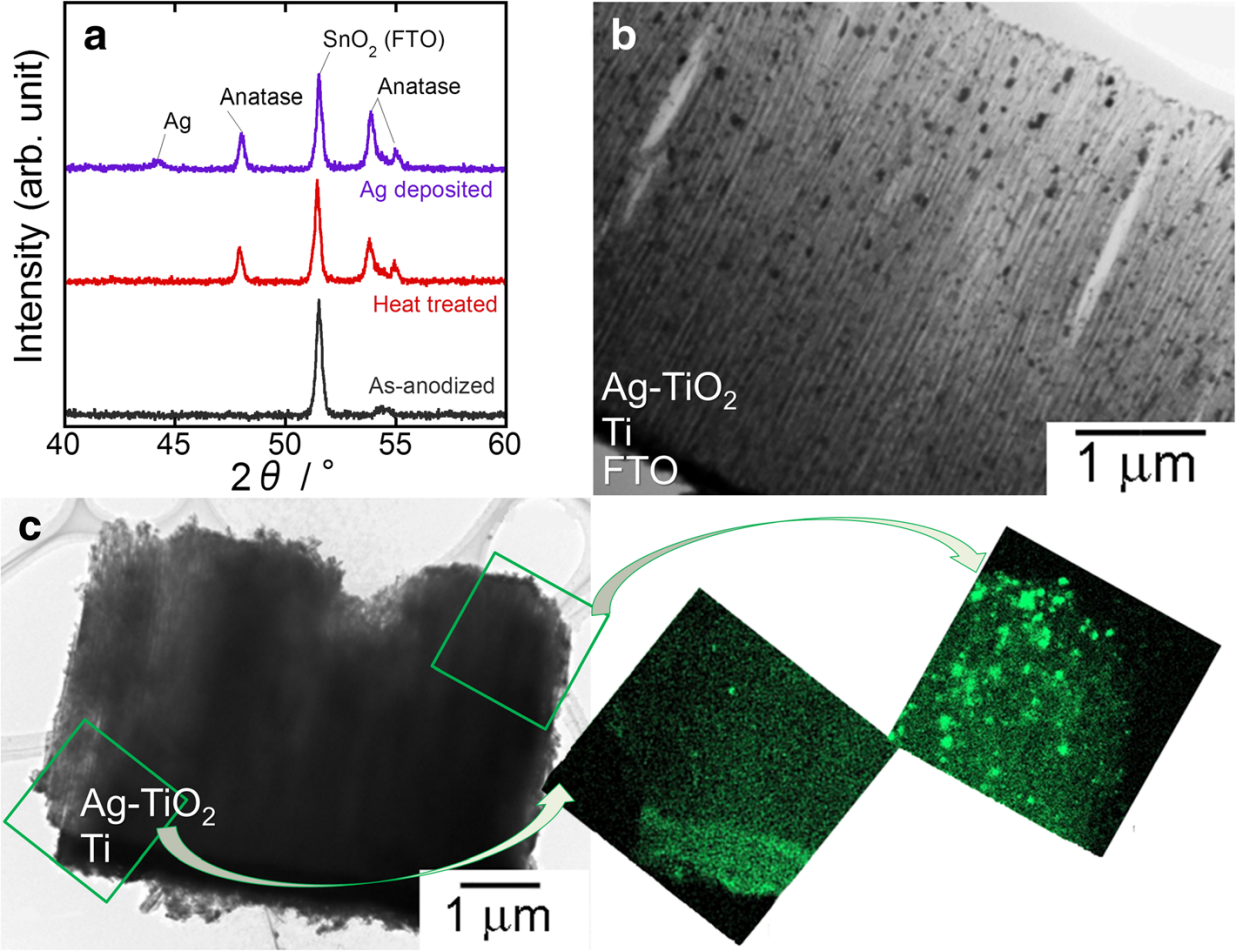
XRD patterns of TNT arrays before and after heat treatment and subsequent Ag nanoparticle deposition (**a**). Cross-sectional TEM (**b**) and EDX mappings associated with the corresponding TEM image (**c**) of Ag nanoparticle deposited-TNT arrays

Figure 4a shows the XRD patterns of TNT arrays on FTO before and after heat treatment and subsequent Ag nanoparticle deposition. Peaks from FTO were only observed in the pattern of as-anodized TNT arrays. On the other hand, the heat-treated sample showed anatase peaks as well as FTO peaks in the XRD pattern. This indicated that amorphous TiO_2_ crystallized to become anatase TiO_2_ through annealing at 450 °C. It was also confirmed that the sheet resistance of FTO was not deteriorated by the heating process. A small peak of Ag appeared at ~44° after Ag nanoparticle deposition process. The dispersion state of deposited Ag was studied using TEM-EDX analyses. The cross-sectional TEM image of Ag nanoparticle-deposited TNT arrays (Fig. 4b) revealed that Ag was deposited as oval nanoparticles with minor axes of 10–50 nm, which were smaller than the pore diameter of TNTs (~50 nm). This means that the nanoparticles were deposited at the inside of the tubular pores of arrays. The TEM-EDX results (Fig. 4c) showed that Ag nanoparticles were preferentially deposited onto the upper part of the arrays, so the bottom part of them was almost empty. This state of Ag distribution was formed presumably because the TNTs were not fully filled with AgNO_3_ solution when Ag was deposited by UV radiation. However, the dispersion state of Ag in this sample is probably suitable to enhance DSSC performance because of the following two reasons: (i) if the arrays were fully covered with Ag, dye molecules could not be adsorbed on TiO_2_; (ii) surface plasmon resonance of Ag nanoparticles shows not only absorption but also scattering of light, thus the bottom part of TNT arrays should not be coated with Ag. Otherwise, certain amount of incident light is scattered by LSPR of Ag nanoparticles and not goes into DSSCs.

**Fig. 5:**
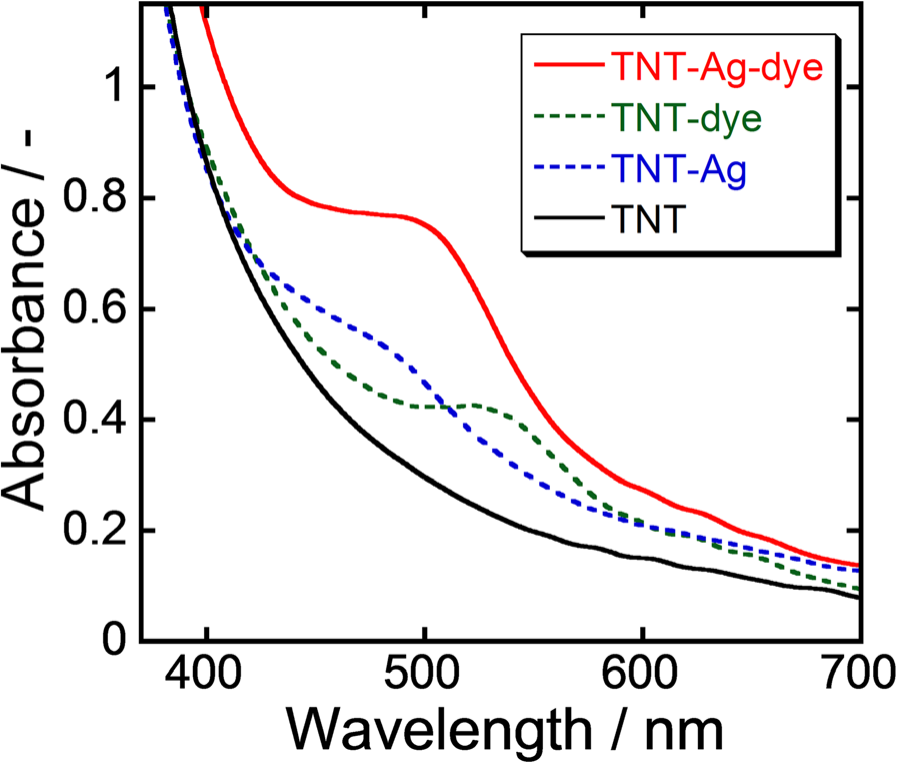
UV-Vis spectra of TNT arrays before and after Ag nanoparticle deposition and dye loading

Figure 5 shows the UV-visible (Vis) spectra of TNT arrays on FTO substrates before and after Ag nanoparticle deposition and dye loading. TNT arrays showed strong absorption in the UV region due to interband transition of anatase. In addition to this absorption, TNT arrays with Ag nanoparticles showed a broad peak centered at 490 nm, which is attributed to LSPR of Ag nanoparticles. TNT arrays adsorbed with dye showed another absorption peak at 520 nm, which is defined as absorption of the dye molecules. TNT arrays with both Ag nanoparticles and dye (TNT-Ag-dye) showed a broad and strong peak centered at 500 nm, which is presumably integrated absorption of Ag nanoparticles and dye. This result indicates that Ag nanoparticle deposition clearly enhances the photon harvesting ability of TNT arrays adsorbed with dye.

### DSSC performance

**Fig. 6:**
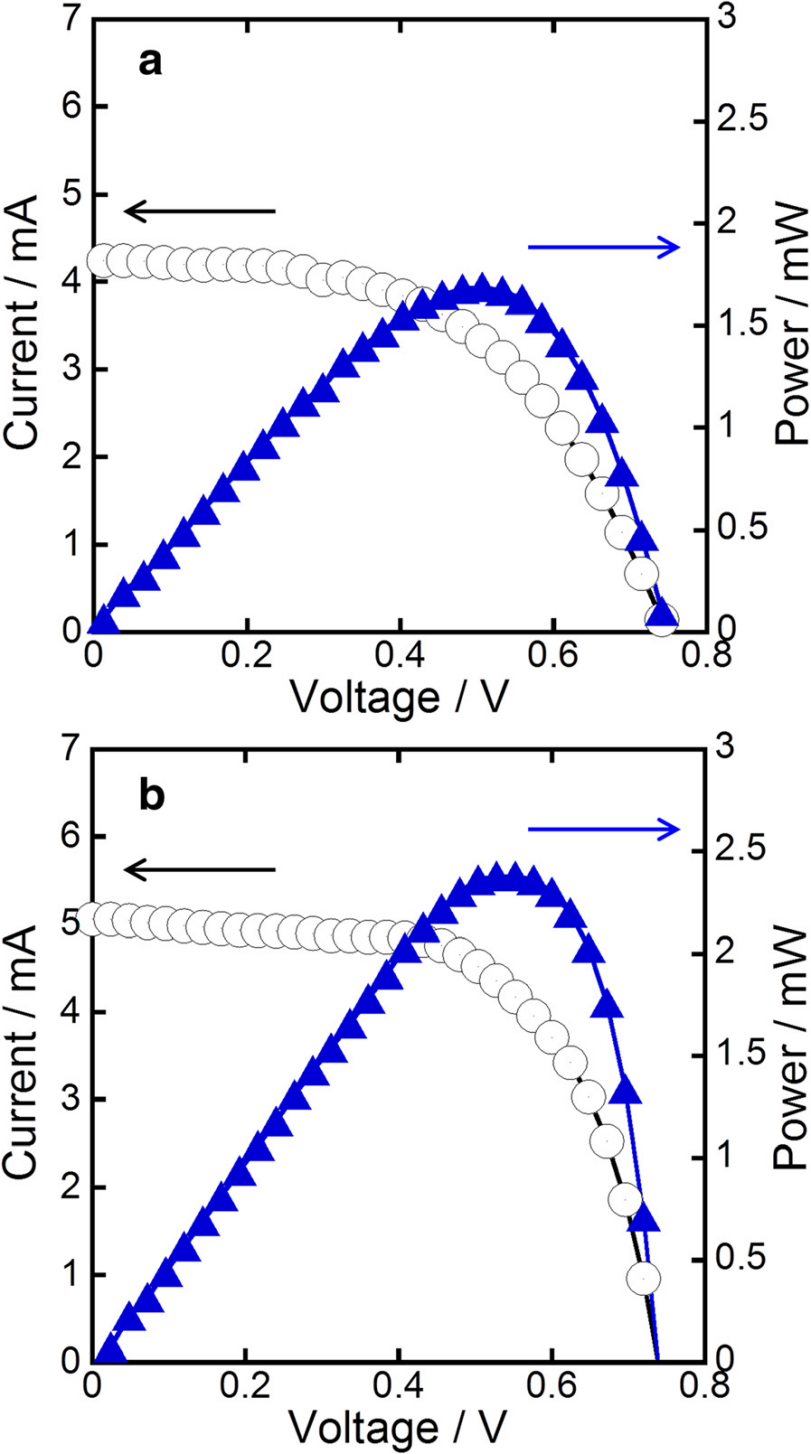
Current-voltage and power-voltage characteristics of DSSC fabricated using TNT arrays without (**a**) and with (**b**) Ag nanoparticles. The blue line (power, *P*) was given by *P* = *I* × *V*

Figure 6a shows the current-voltage characteristics of DSSC fabricated with annealed TNT arrays on FTO. The power conversion efficiency of the DSSC was 1.39 % (open-circuit voltage V_OC_ = 0.73 V, short-circuit density J_SC_ = 4.2 mA cm^−2^, fill factor FF = 0.44). On the other hand, the DSSC fabricated from Ag nanoparticle-deposited TNT arrays yielded a conversion efficiency of 2.03 % (V_OC_ = 0.76 V, J_SC_ = 5.0 mA cm^−2^, FF = 0.54) (Fig. 6b). The improvement of J_SC_ caused by depositing Ag nanoparticles should mainly be due to enhanced light-harvesting ability. LSPR of Ag nanoparticles strongly absorb and scatter photons compared to almost all materials including dyes, thus more photons are captured by the DSSC with Ag nanoparticles [16]. Additionally, since the electric field near Ag nanoparticles becomes very strong when LSPR is induced, the electrons of dyes are effectively excited by the enhanced local field. Therefore, the improved J_SC_ is reasonably explained in terms of the abovementioned LSPR effects, because more excited electrons are generated by depositing Ag nanoparticles on the arrays. The deposition of Ag nanoparticles on TNTs should also have negative effects on DSSC performance, for example, the excited electrons are captured by Ag nanoparticles leading to deteriorated electron conductivity of the TNTs. However, the negative effects were hidden by the positive effect of improved light-harvesting ability. On the other hand, the reason why FF was improved by Ag deposition is still unclear. Presumably, the deposition of Ag nanoparticles occurred predominantly at the defect sites because of the higher surface energy of the sites. Thus, Ag nanoparticles got rid of the defect sites where charge recombination often occurs. Considering that as-purchased N719 was used without further purification process in this work, higher conversion efficiency should be obtainable by using properly purified or recently developed dyes [17]. A surface treatment of TiO_2_ electrode by TiCl_4_, which is often employed to improve J_SC_ and FF [18, 19], was also not carried out in this work. Moreover, optimization of the length of TNT arrays should also be effective for enhancement of our DSSC performance.

## Conclusions

The morphology of TNT arrays on FTO substrates was controlled by altering applied voltage and anodizing duration. Debris-free TNT arrays with a length of 3.9 μm and a pore diameter of 51 nm were successfully prepared and used for subsequent Ag nanoparticle deposition and DSSC fabrication. The deposited Ag was mainly distributed at the upper part of TNT arrays (until ~1 μm from the top), so the bottom part was almost in the absence of Ag. Evaluated DSSC performance unveiled that Ag nanoparticle deposition was effective to increase J_SC_ and FF, while V_OC_ was unaffected. Although the reason why FF was improved was still unclear, the increased J_SC_ was well explained in terms of enhanced light-harvesting ability by LSPR of Ag nanoparticles. Since TiCl_4_ treatment, dye purification, and optimization of Ag loading quantity and TNT length were not carried out in this work, much higher conversion efficiency can be expected in the future.

## Abbreviations

NPs: Nanoparticles
FTO: Fluorine-doped tin oxide
DSSC: Dye-sensitized solar cell
CIGS: Copper indium gallium diselenide
TCO: Transparent conducting oxide
LSPR: Localized surface plasmon resonance
EG: Ethylene glycol
UV: Ultra-violet
SEM: Scanning electron microscope
XRD: X-ray diffractometer
TEM: Transmission electron microscope
EDX: Energy-dispersive X-ray spectroscope
FIB: Focused-ion beam
